# New Books

**Published:** 2004-09

**Biostatistics: A Bayesian Introduction**

George G. Woodworth

Hoboken, NJ:John Wiley & Sons, 2004. 352 pp. ISBN: 0-471-46842-8, $89.95

**Biostatistics and Epidemiology: A Primer for Health and Biomedical Professionals, 3rd ed.**

Sylvia Wassertheil-Smoller

New York:Springer-Verlag, 2004. 243 pp. ISBN: 0-387-40292-6, $34.95

**Burning Season: The Murder of Chico Mendes and the Fight for the Amazon Rain Forest**

Andrew Revkin

Washington, DC:Island Press/Shearwater Books, 2004. 336 pp. ISBN: 1-55963-089-2, $16

**Critical Care Toxicology**

Jeffrey Brent

Burlington, MA:Elsevier, 2004. 1504 pp. ISBN: 0-8151-4387-7, $175

**Dead Heat: Globalization and Global Warming**

Tom Athanasiou, Paul Baer

New York:Seven Stories Press, 2004. 176 pp. ISBN: 1-58322-477-7, $9.95

**Developmental Immunotoxicology**

Steven D. Holladay, ed.

Boca Raton, FL:CRC Press, 2004. 416 pp. ISBN: 0415284570, $139.95

**Earth in Mind—10th Anniversary Edition On Education, Environment, and the Human Prospect**

David W. Orr

Washington, DC:Island Press, 2004. 224 pp. ISBN: 1-55963-495-2, $19.95

**Environmental Challenges in the Mediterranean 2000–2050**

Antonio Marquina, ed.

New York:Kluwer Academic Publishers, 2004. 400 pp. ISBN: 1-4020-1948-3, $72

**Environmental Health: Third Edition**

Dade W Moeller

Cambridge, MA:Harvard University Press, 2004. 560 pp. ISBN: 0-674-01494-4, $65

**Environmental Toxicology: Biological and Health Effects of Pollutants, 2nd ed.**

Ming-Ho Yu

Boca Raton, FL:CRC Press, 2004. 352 pp. ISBN: 1-56670-670-X, $89.95

**Environmentalism and the Technologies of Tomorrow Shaping the Next Industrial Revolution**

Robert Olson, David Rejeski, eds.

Washington, DC:Island Press, 2004. 184 pp. ISBN: 1-55963-769-2, $22.50

**Hot Spot Pollutants: Pharmaceuticals in the Environment**

Daniel Dietrich, Simon Webb, Thomas Petry, J. August, Ferid Murad, Daryl Granner

Burlington, MA:Elsevier, 2004. 350 pp. ISBN: 0-12-032953-0, $159.95

**Immunotoxicology of Drugs and Chemicals**

J. Descotes

Burlington, MA:Elsevier, 2004. 400 pp. ISBN: 0-444-51093-1, $167.50

**Last Refuge: Patriotism, Politics, and the Environment in an Age of Terror**

David W. Orr

Washington, DC:Island Press, 2004. 192 pp. ISBN: 1-55963-528-2, $20

**Molecular Toxicology Protocols**

Phouthone Keohavong, Stephen G Grant

Totowa, NJ:Humana Press, 2004. 550 pp. ISBN: 1-58829-084-0, $99.50

**Ovarian Toxicology**

Patricia B. Hoyer, ed.

Boca Raton, FL:CRC Press, 2004. 256 pp. ISBN: 0-4152-8795-2, $139.95

**Pioneering Research: A Risk Worth Taking**

Donald W. Braben

Hoboken, NJ:John Wiley & Sons, 2004. 208 pp. ISBN: 0-471-48852-6, $39.95

**Plant Toxicology, 4th ed.**

Bertold Hock, Erich F. Elstner

Boca Raton, FL:CRC Press, 2004. 650 pp. ISBN: 0-8247-5323-2, $195

**Predicting Chemical Toxicity and Fate**

Mark T.D. Cronin, David J. Livingstone

Boca Raton, FL:CRC Press, 2004. 472 pp. ISBN: 0-4152-7180-0, $99.95

**Requiem for Nature**

John Terborgh

Washington, DC:Island Press, 2004. 246 pp. ISBN: 1-55963-588-6, $15

**The Dictionary of Gene Technology: Genomics, Transcriptomics, Proteomics, 3rd ed.**

Günter Kahl

Hoboken, NJ:John Wiley & Sons, 2004. 1,300 pp. ISBN: 3-527-30765-6, $230

**The Empty Ocean**

Richard Ellis

Washington, DC:Island Books/Shearwater Books, 2004. 384 pp. ISBN: 1-55963-637-8, $16

**The New Consumers: The Influence of Affluence on the Environment**

Norman Myers, Jennifer Kent

Washington, DC:Island Press, 2004. 192 pp. ISBN: 1-55963-997-0, $24

